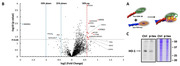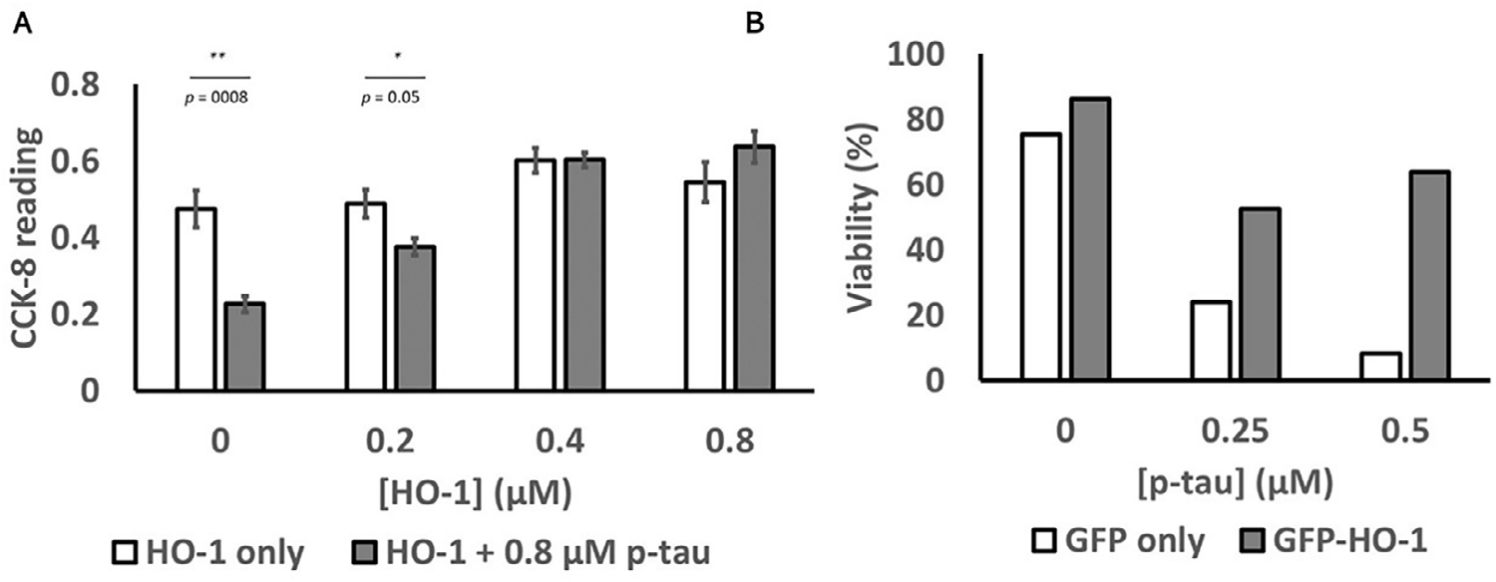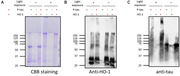# Antagonism between Hyperphosphorylated Tau and Heme Oxygenase‐1 in Alzheimer’s Disease

**DOI:** 10.1002/alz.090017

**Published:** 2025-01-03

**Authors:** Hsiao‐Tien Chien Hagar, Min‐Hao Kuo

**Affiliations:** ^1^ Michigan State University, East Lansing, MI USA

## Abstract

**Background:**

Abnormal phosphorylation of tau is key to Alzheimer’s disease progression. The details of cellular dysfunction or death caused by hyperphosphorylated tau remain unclear. It is crucial to understand the mechanism for drug design. We use our PIMAX system to synthesize hyperphosphorylated tau that causes cell and neuron death at low‐micromolar concentrations. This work reveals the likely relationship between heme oxygenase 1 and pathological tau.

**Methods:**

To understand the molecular underpinnings by which p‐tau kills cells, we used label‐free mass spectrometry to examine the proteomic changes of the SH‐SY5Y neuroblastoma cells after six‐hour treatment with 1 µM of recombinant hyperphosphorylated tau (p‐tau). HO‐1 reduction was among the most prominent alterations instigated by p‐tau. Accordingly, we then tested the hypothesis that HO‐1 deficiency was key to cell death following p‐tau treatment by examining whether supplying extra HO‐1 to cells treated with p‐tau would exhibit resistance. Lastly, we examined the physical interaction between HO‐1 and p‐tau by photo‐crosslinking.

**Result:**

One micromolar of p‐tau causes drastic morphological changes and death of cells after 16 – 20 hours of treatment. Six hours after the addition of p‐tau, SH‐SY5Y cells still maintained normal morphology, but mass spectrometry revealed conspicuous proteomic changes, among which heme oxygenase‐1 (HO‐1) showed the most significant reduction, whereas several other proteins were upregulated under the same condition. The role of HO‐1 in p‐tau mediated cell death was confirmed by the observations that SH‐SY5Y cells exhibited increased resistance to p‐tau if HO‐1 was overexpressed or when cells were co‐treated with recombinant p‐tau and HO‐1 proteins. HO‐1 protects cells likely through a direct interaction with p‐tau because photo‐crosslinking resulted in a high‐molecular‐weight protein complex detectable by both tau and HO‐1 antibodies.

**Conclusion:**

Hyperphosphorylated tau likely physically sequesters HO‐1 as part of the mechanisms of tau pathology. Upregulating HO‐1 confers cellular resistance to hyperphosphorylated tau. Our observations can help the design of reagents targeting the interface between p‐tau and HO‐1. Such reagents may have therapeutic potentials for Alzheimer’s disease and other tauopathies.